# Guided tissue regeneration with heterologous materials in primary subtalar arthrodesis: a case report

**DOI:** 10.1186/s13256-016-0907-6

**Published:** 2016-05-03

**Authors:** Igor Frangez, Tea Kasnik, Matej Cimerman, Dragica Maja Smrke

**Affiliations:** 1 Department of Traumatology, University Medical Center Ljubljana, Zaloska cesta 2, 1000 Ljubljana, Slovenia; 2 Medical Faculty of Ljubljana, Vrazov trg 2, 1000 Ljubljana, Slovenia

**Keywords:** Guided tissue regeneration, Heterologous bone graft, Heterologous collagen membrane, Subtalar arthrodesis

## Abstract

**Background:**

Calcaneal fractures are relatively rare and difficult to treat. Treatment options vary based on the type of fracture and the surgeon’s experiences. In recent years, surgical procedures have increasingly been used due to the better long-term results. We present a case where guided tissue regeneration was performed in a calcaneal fracture that needed primary subtalar arthrodesis. We used the principles of guided tissue regeneration from oral surgery to perform primary subtalar arthrodesis and minimize the risk of non-union. We used a heterologous collagen membrane, which acts as a mechanical barrier and protects the bone graft from the invasion of unwanted cells that could lead to non-union. The collagenous membrane also has osteoconductive properties and is therefore able to increase the osteoblast proliferation rate.

**Case presentation:**

A 62-year-old Caucasian woman sustained multiple fractures of her lower limbs and spine after a fall from a ladder. Her left calcaneus had a comminuted multifragmental fracture (Sanders type IV) with severe destruction of the cartilage of her subtalar joint and depression of the Böhler’s angle. Therefore, we performed primary arthrodesis of her subtalar joint with elevation of the Böhler’s angle using a 7.3 mm titanium screw, a heterologous cortico-cancellous collagenated pre-hydrated bone mix, a heterologous cancellous collagenated bone wedge, and a heterologous collagen membrane (Tecnoss®, Italy). The graft was fully incorporated 12 weeks after the procedure and a year and a half later our patient walks without limping. We present a new use of guided tissue regeneration with heterologous materials that can be used to treat extensive bone defects after bone injuries.

**Conclusions:**

We believe that guided tissue regeneration using heterologous materials, including a heterologous collagen membrane that presents a mechanical barrier between soft tissues and bone as well as a stimulative component that enhances bone formation, could be more often used in bone surgery.

## Background

Calcaneal fractures are relatively rare and difficult to treat. They represent about 2 % of all fractures. Approximately 60–75 % of fractures lie within the joint (intra-articular) [1]. There are many classifications for calcaneal fractures based either on plain radiographs or CT scan. The most used classification is Sander’s system and it divides fractures into four stages, where stage I are all non-displaced fractures and stage IV are fractures with three or more fragments. Treatment options depend upon the stage and can be conservative or surgical. In recent years, surgical procedures have gained more attention [2]. The aim of operative treatment is to reduce the subtalar joint and to restore the anatomical morphology and function of the bone.

Despite many new surgical techniques, the long-term outcomes of patients with comminuted calcaneal fractures remain poor. Over the years, these patients are prone to develop painful posttraumatic joint disease that reduces the quality of life [3]. In such cases, primary subtalar arthrodesis can be used to prevent early arthrosis. However, this procedure is rarely performed and its indications are controversial [4]. Usually it is indicated in patients with severe comminuted calcaneal fractures with destruction of cartilage and severe depression of Böhler’s angle [4].

Many reports have suggested that, despite using different treatments that preserve the joint, subtalar arthrosis almost universally occurs [5]. This has resulted in primary subtalar arthrodesis becoming more common: patients who have undergone primary arthrodesis have better outcomes than patients who retain some motion in the joint [6].

Instead of an autologous corticospongiosus graft from the iliac crest, a heterologous porcine or equine bone graft can be used. To accelerate bone formation, the concept of guided tissue regeneration (GTR) can be applied [7, 8].

GTR, developed in 1976 by Melcher [8], uses the basic principle of the different speed potentials of the cellular components that migrate into the bone defect during the healing process [9]. A mechanical barrier, in our case a collagen resorbable membrane, prevents ingrowth of unwanted cells and allows the passage of desired cells. In the case of bone surgery, osteoprogenitor cells are favorable to the site of the defects [10, 11].

The purpose of this article is to present our results after a primary subtalar arthrodesis was treated with GTR using different heterologous materials. The use of different bone substitutes is already well known in bone surgery, but operative results can be improved by the additional use of a heterologous collagen membrane, which acts as a mechanical barrier that protects the bone graft from the invasion of unwanted cells that could lead to non-union. The collagenous membrane also has osteoconductive properties and is therefore able to increase the osteoblast proliferation rate and enables faster incorporation of the graft.

## Case presentation

A 62-year-old Caucasian woman fell approximately 6 m from a ladder. At our emergency department she was diagnosed with a comminuted dislocated fracture of her left proximal tibia, a proximal fracture of her right tibia, a pilon fracture of her right tibia, a comminuted fracture of her left calcaneus, a fracture of her left lateral malleolus, and an L-1 spinal fracture. We urgently operated, performing external fixation of both tibial fractures and osteosynthesis of her left fibula as well as external fixation of her right pilon fracture.

**Fig. 1:**
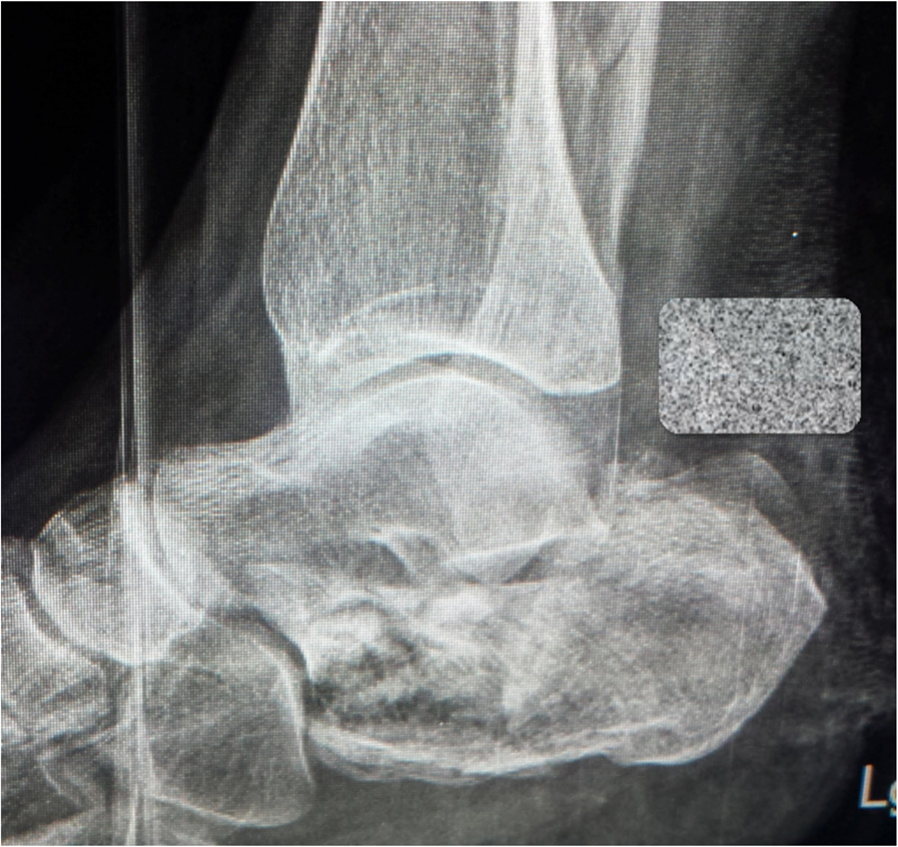
X-ray of calcaneal fracture after injury

Her left calcaneus had a comminuted multifragmental fracture (Sanders type IV) with severe destruction of the cartilage of her subtalar joint and depression of Böhler’s angle (Fig. 1). Considering the severity of the fracture, we decided to perform primary arthrodesis of her subtalar joint with elevation of the Böhler’s angle instead of osteosynthesis of the calcaneus. Because of the severe damage to her soft tissues, we decided to perform the primary arthrodesis after 5 weeks. Because she had multiple fractures and consequently multiple operations, we decided to use a heterologous bone graft instead of the autologous cortico-cancellous graft from her iliac crest to avoid possible additional complications at the donor site.

**Fig. 2:**
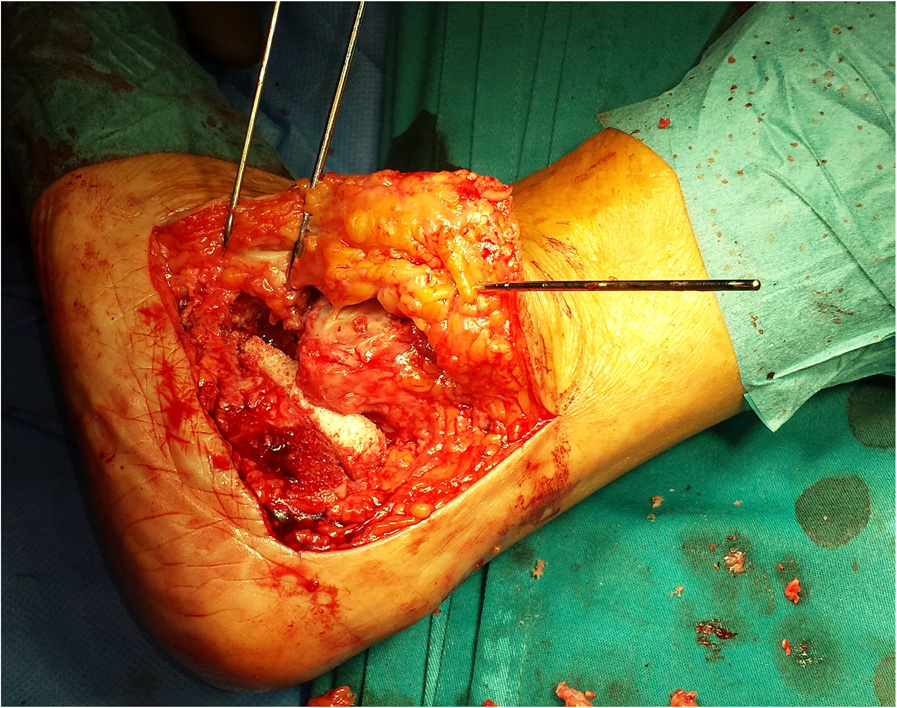
A heterologous cancellous collagenated bone wedge was inserted for restoration of the Böhler’s angle

**Fig. 3:**
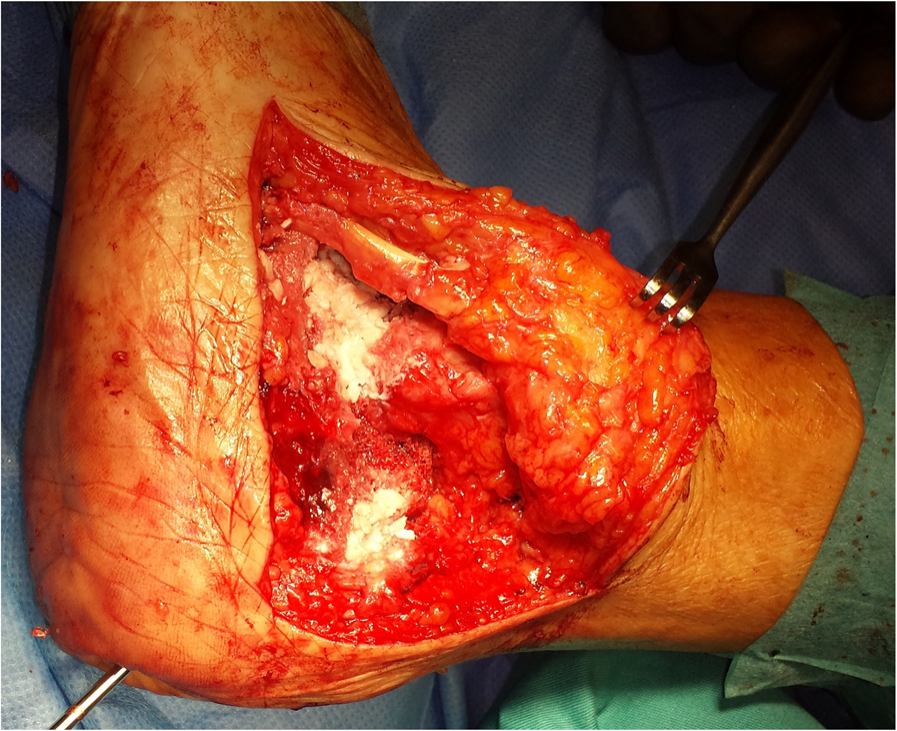
A heterologous cortico-cancellous collagenated pre-hydrated bone mix was used for spongioplasty

**Fig. 4:**
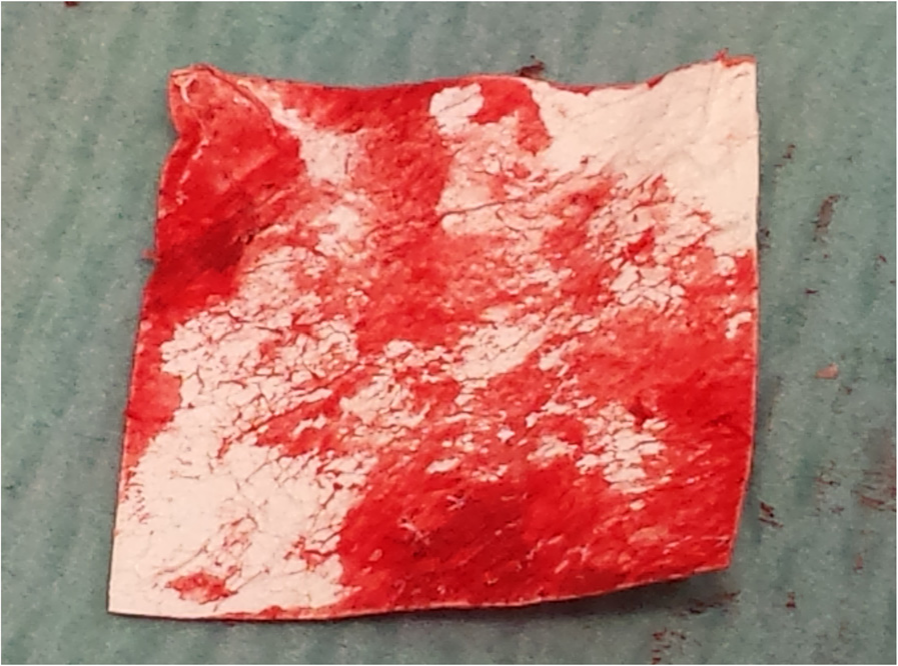
A heterologous collagen membrane was first moistened with our patient’s blood to soften it and allow adjustment to the surface of the operative wound

We used a 7.3 mm titanium screw for the subtalar arthrodesis and a V block (an equine heterologous cancellous collagenated bone wedge; OsteoBiol, Tecnoss®) to restore the Böhler’s angle. This was used instead of an autologous cortico-cancellous graft (Fig. 2). For spongioplasty, we used an equine heterologous cortico-cancellous collagenated pre-hydrated bone mix (mp3, Tecnoss®) (Fig. 3). A resorbable collagen membrane (heterologous collagen membrane, Evolution, Tecnoss®) was used to maintain tissue guidance during regeneration and to avoid potential non-union (Fig. 4). Our patient received antibiotic prophylaxis for 2 days; she was mobilized 1 day after the operation (in a wheelchair due to her other fractures) and drainage was removed on the second day. After 1 week she was sent to our University Medical Rehabilitation Center. She had regular check-ups every second week (with X-rays). Six weeks after her last operation (subtalar arthrodesis on her left side), she was allowed full weight-bearing with crutches on her right foot and partial weight-bearing on her left foot (20–30 kg).

Four weeks after the subtalar arthrodesis, a plain X-ray showed good incorporation of the graft. Eight weeks later, the wound had healed properly and she was allowed to begin full weight-bearing. The graft was fully incorporated 12 weeks after the procedure.

**Fig. 5:**
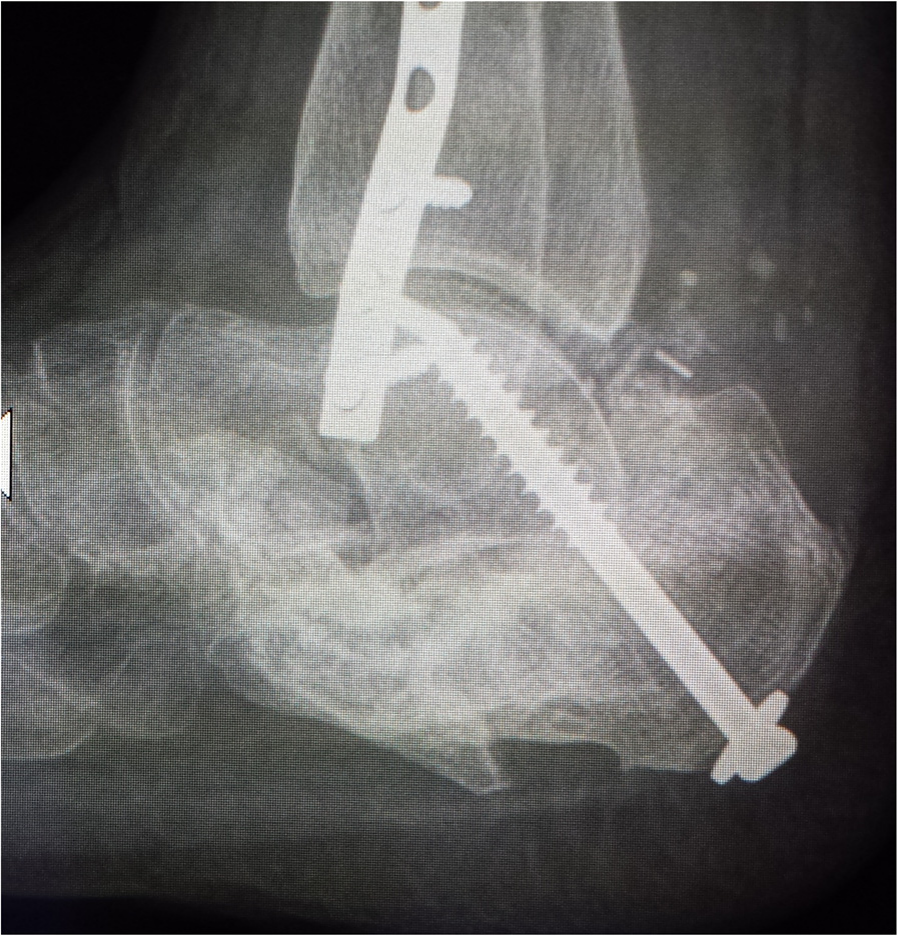
Final result after 1.5 years after operative treatment

A year and a half after her injury, our patient had no pain and walked without limping (Fig. 5).

## Discussion

Calcaneal fractures are difficult to treat, especially when extensive depression of Böhler’s angle is present. Primary subtalar arthrodesis appears to be a good treatment option, because most comminuted calcaneal fractures end in post-traumatic subtalar arthrosis [5]. GTR has been used in oral surgery for many decades and its principles can also be used in orthopedic trauma surgery [9, 12]. In GTR for bone defect fulfillment, different grafts can be used (an autologous or allogenous bone graft, synthetic materials, or heterologous bone) but a main component is a resorbable collagen membrane, which prevents ingrowth of unwanted cells and allows the passage of desired cells. In manufacturing the heterologous bone, the antigenic components are neutralized but the collagen matrix is preserved inside the granules of the biomaterial. Collagen has a key role in the bone regeneration process: (1) it acts as a valid substrate for platelet activation and aggregation; (2) it serves to attract and differentiate the mesenchymal stem cells in the bone marrow; (3) it increases the proliferation rate of osteoblasts by up to three times; and (4) it stimulates the activation of the platelets, osteoblasts, and osteoclasts in the tissue healing process [13, 14].

We present a new application of GTR, using an equine heterologous graft and a resorbable collagen membrane together, which can be used for extensive bone defects after comminuted calcaneal fractures. The main reason we used an equine heterologous bone wedge was the severe depression of the Böhler’s angle in our patient, which would demand a large amount of autologous bone graft.

The described principles of GTR can also be also in cases of secondary arthrodesis, fulfillment of large bone defects in comminuted fractures, and in the case of non-union of the bone.

## Conclusions

A comminuted calcaneal fracture can be difficult to manage and it the approach is largely based on the surgeon’s experience. In our case, where arthrodesis and spongioplasty were needed, we used GTR with a resorbable collagen membrane and an equine heterologous bone graft. The rapid incorporation of the heterologous bone graft, which was enhanced by the collagen membrane, supports the efficacy of the described principles of GTR and we suggest that this approach could be used more often in cases where bone grafts are needed.

## Consent

Written informed consent was obtained from the patient for publication of this case report and accompanying images. A copy of the written consent is available for review by the Editor-in-Chief of this journal.
